# Binding-Induced Folding of a Natively Unstructured Transcription Factor

**DOI:** 10.1371/journal.pcbi.1000060

**Published:** 2008-04-11

**Authors:** Adrian Gustavo Turjanski, J. Silvio Gutkind, Robert B. Best, Gerhard Hummer

**Affiliations:** 1 Oral and Pharyngeal Cancer Branch, National Institute of Dental and Craniofacial Research, National Institutes of Health, Bethesda, Maryland, United States of America; 2 Laboratory of Chemical Physics, National Institute of Diabetes and Digestive and Kidney Diseases, National Institutes of Health, Bethesda, Maryland, United States of America; Harvard University, United States of America

## Abstract

Transcription factors are central components of the intracellular regulatory networks that control gene expression. An increasingly recognized phenomenon among human transcription factors is the formation of structure upon target binding. Here, we study the folding and binding of the pKID domain of CREB to the KIX domain of the co-activator CBP. Our simulations of a topology-based Gō-type model predict a coupled folding and binding mechanism, and the existence of partially bound intermediates. From transition-path and Φ-value analyses, we find that the binding transition state resembles the unstructured state in solution, implying that CREB becomes structured only after committing to binding. A change of structure following binding is reminiscent of an induced-fit mechanism and contrasts with models in which binding occurs to pre-structured conformations that exist in the unbound state at equilibrium. Interestingly, increasing the amount of structure in the unbound pKID reduces the rate of binding, suggesting a “fly-casting”-like process. We find that the inclusion of attractive non-native interactions results in the formation of non-specific encounter complexes that enhance the on-rate of binding, but do not significantly change the binding mechanism. Our study helps explain how being unstructured can confer an advantage in protein target recognition. The simulations are in general agreement with the results of a recently reported nuclear magnetic resonance study, and aid in the interpretation of the experimental binding kinetics.

## Author Summary

Protein-protein interactions are central to many physiological processes. Traditionally, the atomic structure of the isolated proteins, as obtained from X-ray crystallography or NMR spectroscopy, has been thought to determine the molecular recognition and binding process. However, this view has been challenged by the discovery of natively unstructured proteins, including many human transcription factors, which assume ordered molecular structures only upon binding to their targets. Understanding these transitions from a dissociated and unfolded state to a bound and folded state is key to unraveling how transcription factors, proteins involved in the system that controls the transcription of genetic information from DNA to RNA, perform their function. We conducted molecular simulations to study the binding of a well characterized transcription/co-transcription factor complex. We found that the transcription factor is unstructured when binding to its partner, with folding into an ordered structure occurring only after the initial binding event. The resulting coupled folding-and-binding mechanism is found to be in accord with state-of-the-art experimental data. Transcription-factor binding in an unstructured state may confer an advantage in protein target recognition by accelerating the rate of association without compromising the ability to bind to a diverse set of proteins with high specificity and yet relatively low affinity.

## Introduction

A central tenet of genomics is that sequence determines structure, and structure determines function. Even though this is true for many proteins, where structure and function are closely related, a rapidly increasing number of proteins have been found that are natively unfolded or that have long unstructured regions (>50 residues) [1]–[4]. For many of these proteins, and transcription factors in particular [2], this intrinsic disorder appears to be required for their function. Transcription factors are essential players in signal transduction by regulating gene expression in the nucleus in response to changes in the cellular environment. They are regulated themselves by post-transcriptional modifications, for example phosphorylation [5],[6] or ubiquitination. To transmit the correct signal, transcription factors need to associate with many diverse proteins with high specificity and yet relatively low affinity, as they need to dissociate upon signal completion. Having substantial unstructured regions may help in binding to different targets with large surfaces of interaction, and with different conformations, to achieve the required specificity and relative affinity. Moreover, being unstructured may be relevant for the rapid degradation of transcription factors [7].

**Figure 1:**
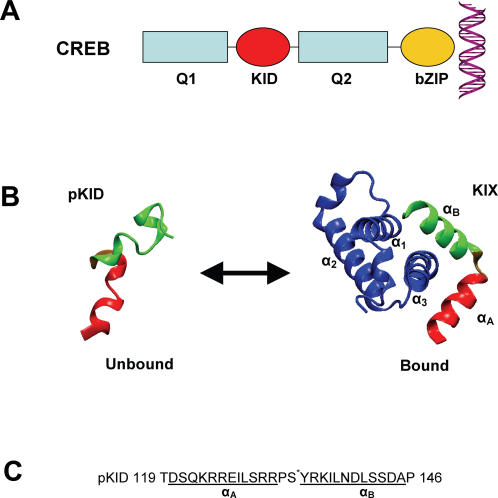
Structure of the pKID/KIX complex. (A) Domain structure of CREB, consisting of DNA-binding domain basic region/leucine zipper (bZIP), the cAMP activated domain KID, and the glutamine-rich domains Q1 and Q2. The sequence of the structured part of pKID is shown with residues in helices α_A_ and α_B_ underlined. (B) Folding and binding of pKID (red: helix α_A_; green: helix α_B_) to the KIX domain of CBP (blue).

The transcription factor cAMP response-element binding protein (CREB) represents a well-characterized paradigm for an unstructured peptide that adopts a folded structure upon binding its co-activator CREB binding protein (CBP) [1],[8]. CREB participates in diverse cellular processes, ranging from glucose homeostasis, survival and proliferation to learning and memory [9],[10]. CREB is one of the best characterized transcription factors in terms of its structure and function [9]–[15]. Like many transcription factors, CREB consists of multiple domains (Figure 1A): (i) a C-terminal basic region/leucine zipper (bZIP) dimerization domain that binds DNA with high specificity for the conserved cAMP-responsive element (CRE) TGACGTCA [16],[17]; (ii) a 60-amino-acid kinase-inducible domain (KID) (amino acids 100–160); and (iii) two hydrophobic glutamine-rich domains, designated Q1 and Q2, that function as constitutive transcriptional activators [10]. CREB has both basal and inducible transcriptional activity [14], the latter being triggered by phosphorylation of the KID domain at Ser^133^ by the protein kinase A (PKA). Indeed, CREB was the first transcription factor whose activity was shown to be tightly regulated by phosphorylation/dephosphorylation [18]. The phosphorylated KID domain (pKID) promotes the recruitment of the transcriptional co-activator CBP (and the related molecule p300) by binding to its kinase-induced domain interacting domain (KIX) [12],[19]. Upon binding to KIX, pKID undergoes a transition from unstructured to structured [12].

The nuclear magnetic resonance (NMR) structure of the pKID/KIX complex indicates that only residues 119 to 146 of pKID are folded [12], forming a two-helix structure with helices α_A_ and α_B_ almost perpendicular to one another. The amphipathic α_B_ helix provides most of the intermolecular contact surface in the complex. Pro^132^ is located in the hinge between the two helices. Phospho-Ser^133^, positioned at the beginning of the α_B_ helix, stabilizes the complex by interacting with Tyr^658^ and Lys^662^ of KIX [12]. NMR experiments have also shown that the unbound pKID domain is mostly unstructured except for the α_A_ helix which contains a significant fraction of helix (>50%), while the helical content in the α_B_ helix is very small (10–15%) [13]. The KIX domain, comprising residues 586–672 of CBP, folds into a three-helix structure with helices α_1_ and α_3_ arranged in parallel, forming a hydrophobic groove that forms the major binding interface for the amphipathic α_B_ helix of pKID.

Phosphorylation of Ser^133^ in KID has been shown to increase the free energy of binding by ∼1.5–3.0 kcal/mol [11],[20],[21], mostly by forming a hydrogen bond with Tyr^658^ of KIX. Although a small increase in the pKID secondary structure has been detected upon phosphorylation, it does not seem to have an important role in the binding process [13]. However, it has been suggested that phosphorylation may promote the formation of a transient structural element similar to the bound conformation, which may thus play an extra role in the binding mechanism [22].

As we completed this work, a related NMR study was published on the mechanism of folding and binding of CREB to CBP [23] where it was suggested that pKID becomes structured only after committing to binding. The proposed mechanism involves the formation of transient encounter complexes that evolve to a single intermediate and then to the high-affinity complex [23]. Encounter complexes have been shown to play a key role during protein-protein association for globular proteins for quite some time [24],[25] and a structural characterization by NMR has recently been reported [26]. Transient complexes in globular proteins are dominated by long-range electrostatic interactions [24],[25],[27], however in the pKID/KIX complex Sugase et. al [23] propose a predominant role for non-specific hydrophobic contacts.

Here, we study the folding and binding mechanism of pKID to KIX by simulation of a Gō-type topology-based model based on the native structure of the pKID:KIX complex. We analyze the binding mechanism quantitatively by identifying the key features of binding transition states (TS) using a Bayesian formula relating the equilibrium and transition-path ensembles [28],[29]. Our simulations are in general agreement with recent NMR data [23] and also help refine the interpretation of the experimental results. We show that this process occurs via a coupled folding and binding mechanism that typically begins with the binding of an unstructured α_B_ region in pKID, followed by the folding of α_B_ into a helix, and finally the docking of the α_A_ helix. Our simulations show that the TS structures of this coupled binding-and-folding reaction are more closely related to the unstructured conformations of pKID than to the bound conformation. Surprisingly, simulations in which the helical propensity of the unbound pKID was increased resulted in a decrease in the rate of binding in comparison to the unstructured pKID. These findings are in contrast to models that invoke the binding of pre-folded conformations or a TS similar to the complex structure. By calculating Φ values from tertiary contacts we have identified a key residue for the coupled folding-and-binding mechanism, Leu^141^, that interacts with the hydrophobic groove of KIX in the TS. Moreover, by adding non-native interactions in addition to those present in the Gō-like model we explored the role of non-specific interactions in the binding process. The simulations of the resulting non-Gō model show an enhanced formation of encounter complexes, in agreement with a recent NMR study [23]. We find that additional sequence-based (“non-native”) attractive interactions do not significantly change the binding mechanism, as captured by the transition state ensemble and the pathways taken between unbound and bound states. However, strengthening the non-native interactions and the resulting formation of non-specific encounter complexes increases the binding rate constant, reflecting the increased interaction cross section, consistent with results for binding of globular proteins and protein folding kinetics [27],[30]. Overall, using a simple coarse-grained model we provide a detailed dynamic picture of the folding and binding process of an unstructured protein that is in accord with state-of-the-art experimental data.

## Results/Discussion

### Coupled Folding and Binding Mechanism

**Figure 2:**
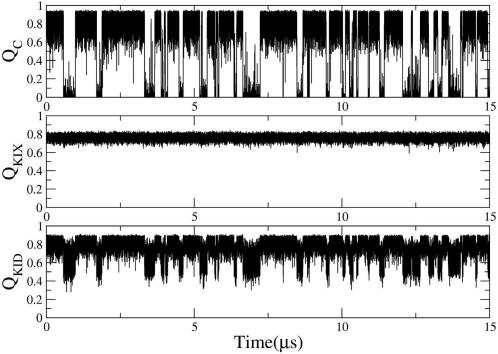
The fraction of native contacts for pKID, KIX, and the complex. Fraction of native amino-acid contacts as a function of time for the intermolecular complex (Q_C_; top), KIX (Q_KIX_; center), and pKID (Q_KID_; bottom). Data are shown for one out of thirteen simulations of the same length.

Figure 1A shows a schematic representation of the distinct domains within CREB, including Q1, Q2, BZIP and pKID as described above. Long equilibrium simulations of the complex were initiated from the NMR structure of the complex (PDB code 1KDX [12]) as depicted in Figure 1B. Only residues 119–146 of phosphorylated KID, here referred to as pKID, undergo folding and binding (Figure 1C). To monitor folding and binding of pKID to KIX separately, we calculated the fraction of intramolecular native amino-acid contacts of KIX (Q_KIX_) and pKID (Q_KID_), and the fraction of intermolecular native contacts (Q_C_) that are formed over the course of the simulation (Figure 2). During most of the simulation time the conformation of KIX remained folded and close to the native structure, held together by an extensive network of inter-residue interactions (as compared to the relatively few interactions between pKID and KIX, which makes it energetically much easier to dissociate the complex than to unfold KIX). However, a minor population of a partially unfolded state was found for the KIX domain (data not shown). This observation is qualitatively consistent with the existence of a partially unfolded high-energy state of KIX that is sparsely populated under native conditions [31]. The conformation of unbound pKID retains part of the intramolecular native contacts, as measured by Q_KID_, mainly because helix α_A_ remains partially folded, in agreement with previous experimental results [13]. The increased stability of helix α_A_ in comparison to helix α_B_ can be explained energetically, from α_A_ having more and stronger intramolecular contacts than α_B_. The fraction of native intermolecular contacts between pKID and KIX, Q_C_, allows us to monitor the exchange at equilibrium between the two most populated states of the system, the bound (Q_C_∼0.9) and unbound conformations (Q_C_∼0.0). The aggregate simulation data include a total of 889 binding and dissociation transitions, allowing us to characterize in detail the dynamic changes that occur during the folding/binding process.

**Figure 3:**
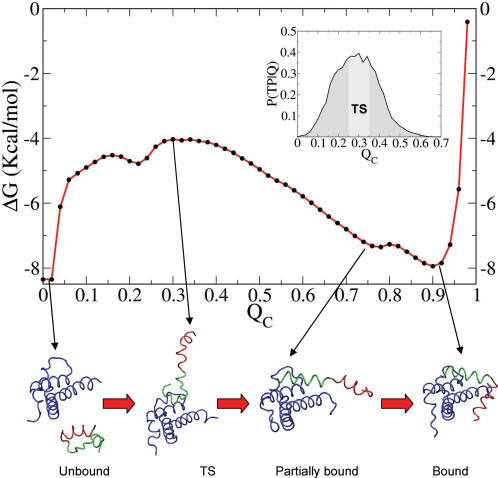
Free energy surface of the folding and binding process as function of Q_C_. Free energy as a function of the fraction of native intermolecular residue-residue contacts Q_C_. Representative conformations along the reaction coordinate are shown (blue: KIX, red: pKID-α_A_, and green: pKID-α_B_). Inset: The probability p(TP|Q_C_) of being on a transition path for a given value of Q_C_. The TS region is marked in light gray.

**Figure 4:**
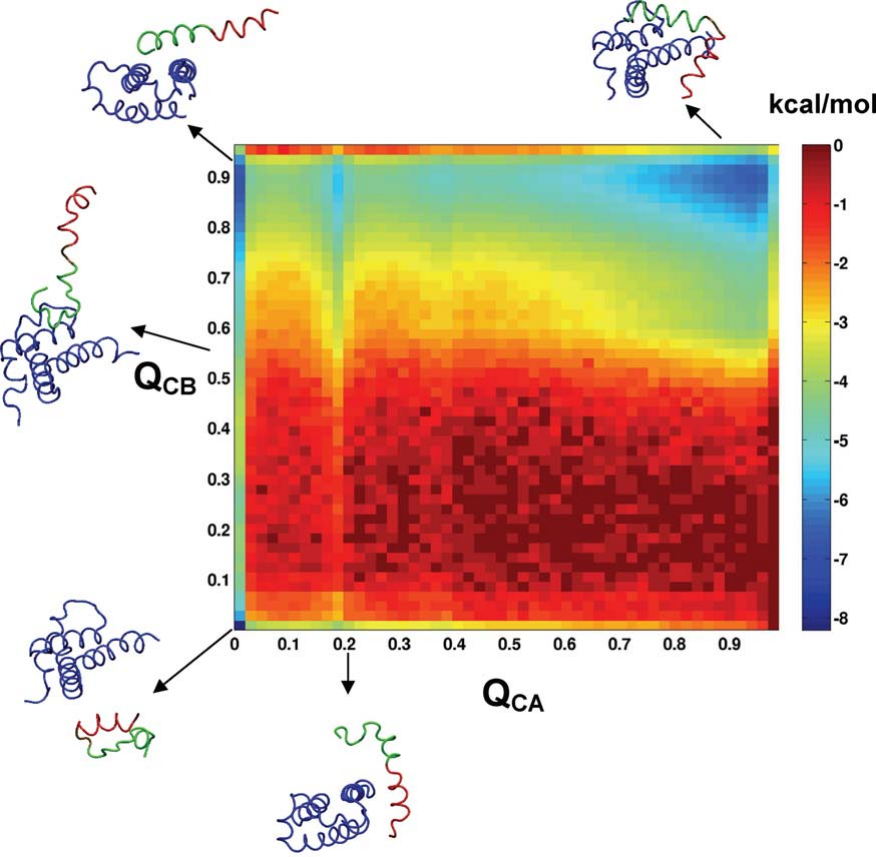
2D free energy surface for the binding-induced folding of pKID. Potential of mean force for binding as a function of the fraction of intermolecular native contacts between helix α_A_ (Q_CA_) and helix α_B_ (Q_CB_) of pKID and KIX. The black line depicts one representative transition path from unbound to bound. Representative structures are shown for important regions of the free energy landscape.

To characterize the binding mechanism of CREB to CBP, we calculated the free energy profile along Q_C_ (Figure 3), which shows a free energy barrier to binding of ∼4 kcal/mol. Notably, the free energy profile also suggests two major populated conformations in the bound state, one with the complex fully formed (Q_C_∼0.9), and a second, “partially bound” intermediate conformation (Q_C_∼0.75). We can gain further insight into the binding mechanism by separating the intermolecular contact fraction, Q_C_, into contacts between KIX and helices α_A_ and α_B_ of pKID, Q_CA_ and Q_CB_, respectively. The binding free energy surface as a function of these two coordinates (Figure 4; representative structures superimposed) shows a dominant L-shaped path from unbound (Q_CA_∼Q_CB_∼0) to fully bound (Q_CA_∼Q_CB_∼0.9), with the contacts to helix α_B_ formed before those to α_A_. High values of Q_CB_ are observed at values of Q_CA_ as low as 0.0–0.2; in contrast, high values of Q_CA_ are not observed in the absence of high Q_CB_. Two types of partially bound intermediates are evident in Figure 4: an intermediate I_A_ with Q_CA_<0.1 and Q_CB_>0.4, and an intermediate I_B_ with Q_CA_>0.1 and Q_CB_<0.1 (with a shallow free energy minimum at Q_CA_∼0.2 and Q_CB_∼0). In the high-population intermediate I_A_, α_B_ is nearly completely bound while α_A_ mostly detaches from KIX. In the low-population intermediate I_B_, α_A_ is partially bound, while α_B_ is detached. The local minimum at Q_CA_∼0.2 and Q_CB_∼0.9 corresponds to structures with transient native interactions formed between helix α_A_ and KIX, with helix α_B_ bound and folded.

It is not entirely surprising that binding is dominated by α_B_, considering that only a small fraction of the intermolecular contacts are formed between α_A_ and KIX (5 out of 32, according to the contact definition in our topological model). The tighter binding between helix α_B_ and KIX seen here is consistent with previous mutational experiments aimed at destabilizing the interaction between helix α_B_ and pKID. When Arg^135^ and Lys^136^ in helix α_B_ were mutated to Gly the binding affinity was found to decrease [11]. However, no significant change in the binding affinity was found upon mutation of Arg^125^ and Glu^126^ to Gly in helix α_A_, suggesting that formation of a helical structure in the α_A_ region contributes relatively little to the overall binding affinity [11].

To gain further insight into the binding mechanism we performed additional simulations using non-Gō potentials. In those simulations, we added sequence-based attractive interactions between non-native residue pairs of pKID and KIX to the otherwise unchanged native-topology based interactions. We explored a broad range of non-native interaction strengths, from 0 to 100% relative to the native interactions (whose strength was not varied). We found that for non-native interaction strengths >50%, the folding and binding process was very “glassy”, governed by escape from many deep local minima in the energy surface (data not shown), in contrast to the facile kinetics deduced from experiments [23]. For non-native interaction strengths between 0 to 50% we obtained similar binding free energy surfaces as with the Gō-type model, with only a small reweighting of the minima (e.g., an increase in complexes formed only by helix α_A_; Figure S1). As the main effect of adding non-native interactions, we find increased flux on a secondary pathway of the folding and binding transition. This pathway involves the formation of intermediate I_B_, from the unbound state to the minimum at Q_CA_∼0.2, Q_CB_∼0, proceeding to the minimum at Q_CA_∼0.2 Q_CB_∼0.9, and then to the bound state (Figure 4 and Figure S1). Whereas the relative contribution of this pathway to the overall reaction is negligible with only native attractions, it is found to have appreciable flux with non-native interactions added, but still much less than the main pathway involving the initial formation of intermediate I_A_.

In summary, the following predominant binding mechanism emerges. Before binding helix α_A_ is mostly formed. Helix α_B_, in contrast, is largely unfolded. Initially, pKID binds to KIX with a largely unstructured α_B_ region that then folds over the surface of KIX. Once helix α_B_ is mostly folded and bound (I_A_), binding of helix α_A_ locks the complex. A secondary pathway with much lower flux, involves the binding of helix α_A_ to the α_3_ helix of KIX, with α_B_ unbound and unstructured (I_B_), folding by the binding of the the initially unstructured α_B_ and its folding. Such independent dissociation of the two pKID helices in the two intermediates also seems reasonable in view of the bound structure (Figure 1B), which shows that the two bound helices lack tertiary interactions with each other, explaining their non-cooperative dissociation.

**Figure 5:**
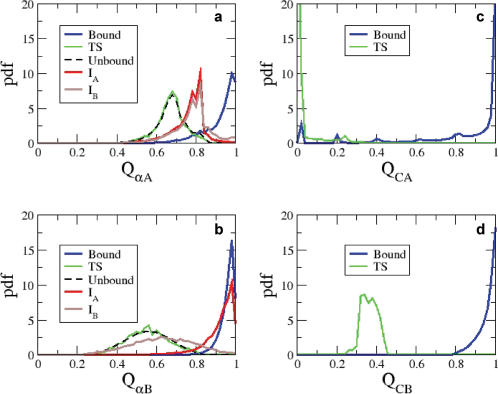
Structural characteristics of the transition state ensemble. (A) and (B) show the probability density function (pdf) of intramolecular native contacts of helix α_A_ (Q_αA_) and helix α_B_ (Q_αB_) of pKID for the unbound state, TS, intermediates I_A_ and I_B_, and bound states. (C) and (D) show the probability density functions of native contact fractions Q_CA_ and Q_CB_ between KIX and helices α_A_ and α_B_ of pKID, respectively, in the bound and transition states.

A recently published study used NMR relaxation-dispersion experiments [23] to infer the formation of a single binding intermediate. Interestingly, the rates of interconversion of the intermediate and the bound state were fitted separately to the relaxation-dispersion curves for certain “clusters” of residues, with marked differences between the rate coefficients for clusters belonging to α_A_ and those belonging to α_B_ (see Table 1 in [23]). The rate of conversion from the intermediate state to the fully-bound state was more than four times faster for clusters of residues belonging to α_B_ than for those belonging to α_A_, whereas the rate for conversion from the fully-bound to the intermediate state was faster for residues in α_A_ than α_B_, an unexpected result for a strictly two-state system. Our simulations suggest a simple mechanism which explains this finding: the intermediate state actually consists of two sub-states, one (I_A_) with helix α_B_ folded and bound, and helix α_A_ unbound and only partially formed; the other (I_B_) with α_A_ bound, and α_B_ unbound. One can associate the rates from helix α_A_ with the interconversion of intermediate I_A_ with the bound state, and those from helix α_B_ with the interconversion of I_B_ and the bound state. From the ratio of these experimentally measured rates (Table 1 [23]), it follows that in steady state the population of I_A_ is about 20 times larger than that of I_B_. Indeed, we find in the simulations that the population of I_A_ is significantly larger than that of I_B_ (Figure 4 and Figure S1), with a population ratio of ∼70, thus differing by a factor of about 3.5 from experiment (or about *k_B_T* ln 3.5∼0.75 kcal/mol in the free energy). Structurally, based on Figure 5A and 5B, we predict ∼80% intramolecular helical contacts for residues on á_A_, and ∼70% for á_B_. This is in reasonable agreement with the experimental estimates of ∼90% folded for á_A_ and ∼70% folded for á_B_, inferred from NMR chemical shift differences [23]. (Note that the measured chemical shift differences of the different residues report on the structure in the respective intermediates: I_A_ for residues in α_A_, and I_B_ in α_B_, not the population-weighted average.) Overall, we find nearly quantitative agreement with experiment, with respect to both the equilibrium populations of the two intermediates and their structural characteristics. Thus our work is a true prediction of binding mechanism from simulation.

### Transition-State Ensemble

To quantify the structural properties of the binding TS, we use the fraction of intermolecular contacts, Q_C_. To show that Q_C_ properly identifies the TS ensemble of the coupled folding-and-binding transition, we apply a Bayesian formalism [28],[29] and compute the conditional probability p(TP|Q_C_) of being on a transition path (TP) given a particular fraction of intermolecular contacts, Q_C_. Transition paths are defined as trajectory segments that connect unbound conformations (Q_C_<0.02) with bound or partially bound conformations (Q_C_>0.7), and vice versa, without re-crossings. The value of Q_C_ with the highest p(TP|Q_C_) is most indicative of being on a transition path, and is used to identify the most reactive states, or “transition states”. The inset in Figure 3 shows that this largest value of p(TP|Q_C_) is obtained for Q_C_∼0.3 for which p(TP| Q_C_)∼0.4. With p(TP|Q_C_) being close to the theoretical maximum of 0.5 for a perfect reaction coordinate of a diffusive process, we conclude that Q_C_ is a relevant coordinate to describe the coupled folding-and-binding transition. We note, that for inertia-dominated processes, the theoretical maximum (in phase space) would be one; however, diffusion is expected to be relevant here for a process in condensed phase that involves the motions of large groups of atoms and solvent. We note further that, with Q_C_ being a good reaction coordinate, as deduced from the high value of p(TP|Q_C_) at the transition state, and with dynamic corrections being small, the maximum of the free energy along Q_C_ coincides nicely with the maximum of p(TP|Q_C_), leading to equivalent transition-state ensembles.

We selected all conformations from transition paths with Q_C_ values in the interval [0.25;0.35] as representatives of the TS ensemble. To characterize the TS structures and relate them to the bound and unbound states, we compared the fractions of native intramolecular contacts of helices α_A_ and α_B_ of pKID (Q_αA,_ Q_αB_) in the bound (Q_C_>0.80) and unbound conformations (Q_C_<0.02), the partially-bound intermediates I_A_ and I_B_, and the TS ensemble (Figure 5A and 5B). In the unbound state helix α_A_ has a smaller fraction of native contacts (maximum at Q_αA_∼0.65) than in the bound state (maximum at Q_αA_∼0.95), consistent with a partial unfolding of helix α_A_ in the I_A_ intermediate. Interestingly, our simulations show that at the TS the structures resemble the unbound conformation, with helix α_A_ partially folded, and helix α_B_ mostly unfolded. At the TS, the distributions of the intramolecular contact fractions Q_αA_ and Q_αB_ are nearly identical to the respective curves for the unbound state (Figure 5A and 5B), and substantially different from the bound ones.

We also calculated the fractions of intermolecular contacts made by each helix (Q_CA_, Q_CB_) in the TS and bound states (Figure 5C and 5D). The distributions of Q_CA_ and Q_CB_ indicate that at the TS helix α_B_ is partially bound to KIX, with α_A_ mostly dissociated. A representative conformation of the TS structure is shown in Figure 3, in which an unstructured helix α_B_ is bound to the hydrophobic groove formed between helices α_1_ and α_3_ on KIX. However, we emphasize that, because of the unstructured nature of pKID in the TS, the TS is best described by a broadly distributed ensemble of conformations in which pKID binds to KIX in different conformations.

### Binding Kinetics of Structured and Unstructured pKID

Our results suggest that binding occurs with the α_B_ helix being largely unstructured. Based on this observation, one would expect little or no effect of α_B_ stability on the kinetics of association, as measured by the on-rate (*k_on_*). We investigated the effect of increased helical structure in the unbound state by conducting simulations in which we strengthened the intramolecular contacts of pKID (Q_KID_) to form pre-folded unbound peptides. By starting from different unbound configurations (Q_C_∼0.0, Q_KID_∼1) randomly chosen from an equilibrium simulation with the original energy function, we performed 350 binding simulations with enhanced intra-helix interactions. The on-rate for the resulting pre-structured helix, *k_on_*(Q_KID_∼1), estimated from mean first passage times, is in fact lower than the on rate for the original simulations by a factor ∼1.6; remarkably, this value is in the same range as theoretical predictions for a “fly-casting” mechanism [32]. In previous experimental studies, the role of secondary structure formation in the folding/binding process was explored through extensive mutations in the α_B_ helix [11],[33]. As stated above, mutations that destabilize the α_B_ helix of pKID decrease the binding affinity; however, mutations intended to increase the α_B_ helix propensity do not enhance binding towards KIX. In some cases, even a reduction in binding has been observed. These results can be rationalized based on our simulations. With the observed unstructured TS of the folding/binding reaction, destabilization of the α_B_ helix should increase the rate of dissociation, *k_off_*, with not significant changes in *k_on_*; in contrast, stabilization of α_B_ should produce at least partially compensating effects, diminishing *k_on_,* by reducing the “fly-casting” effect, and *k_off_*, by stabilizing the bound state, and therefore resulting in only small changes of the overall binding affinity, as observed experimentally [11],[33]. To explore further the role of the α_B_ helix, measurements of the effects of mutations in pKID on the kinetics of association (*k_on_*) and dissociation (*k_off_*) might be insightful.

### Φ Values for pKID-KIX Binding

**Figure 6:**
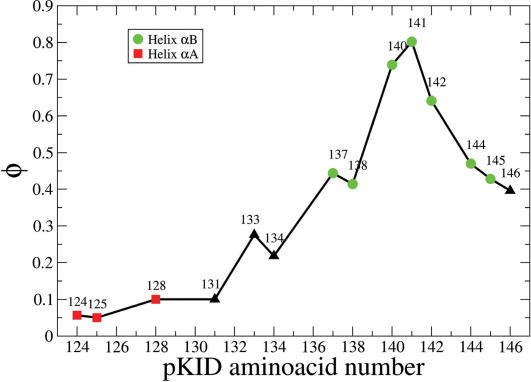
Φ value analysis for the transition state. Φ values for the folding/binding transition state for the residues of pKID forming native contacts with KIX. Red squares indicate amino acids belonging to helix α_A_, green circles indicate amino acids belonging to helix α_B_, and black triangles show Φ values for residues without secondary structure.

In analogy to protein folding [34],[35] we further characterized the TS by calculating Φ values for each amino acid residue of pKID that is involved in native contacts with KIX. We estimated Φ values by using a simple definition in which only native contacts are counted, Φ = Q_xc_(TS)/Q_xc_(bound) [34],[36],[37], where Q_xc_ is the number of native contacts in the respective state between protein 1 and residue *x* of protein 2. Φ values have been extensively used for determining key residues in the folding of proteins and remarkable agreement has been found between theoretical and experimental studies [34],[38],[39]. Φ-value analysis should quantify the amount of intermolecular structure formed at each site in the transition state. The calculated Φ values for the coupled folding-and-binding transition of pKID are shown in Figure 6. According to our analysis, only residues of helix α_B_ seem to be important for TS formation, with the highest Φ values being found for Leu^141^ and the neighboring amino acids Asp^140^ and Ser^142^, implying that hydrophobic interactions involving amino acids at the center of the α_B_ region govern the association mechanism. A critical role for Leu^141^, which is deeply bound into the hydrophobic groove of KIX, has been suggested based on the results of mutating Tyr^650^ of CBP which is located in the wall of the hydrophobic groove [11]. Mutation to Ala resulted in a ∼12-fold decrease in the binding constant [11]. Our study suggests that corresponding kinetic measurements on this mutant should be insightful.

### Effect of Non-Native Interactions

**Figure 7:**
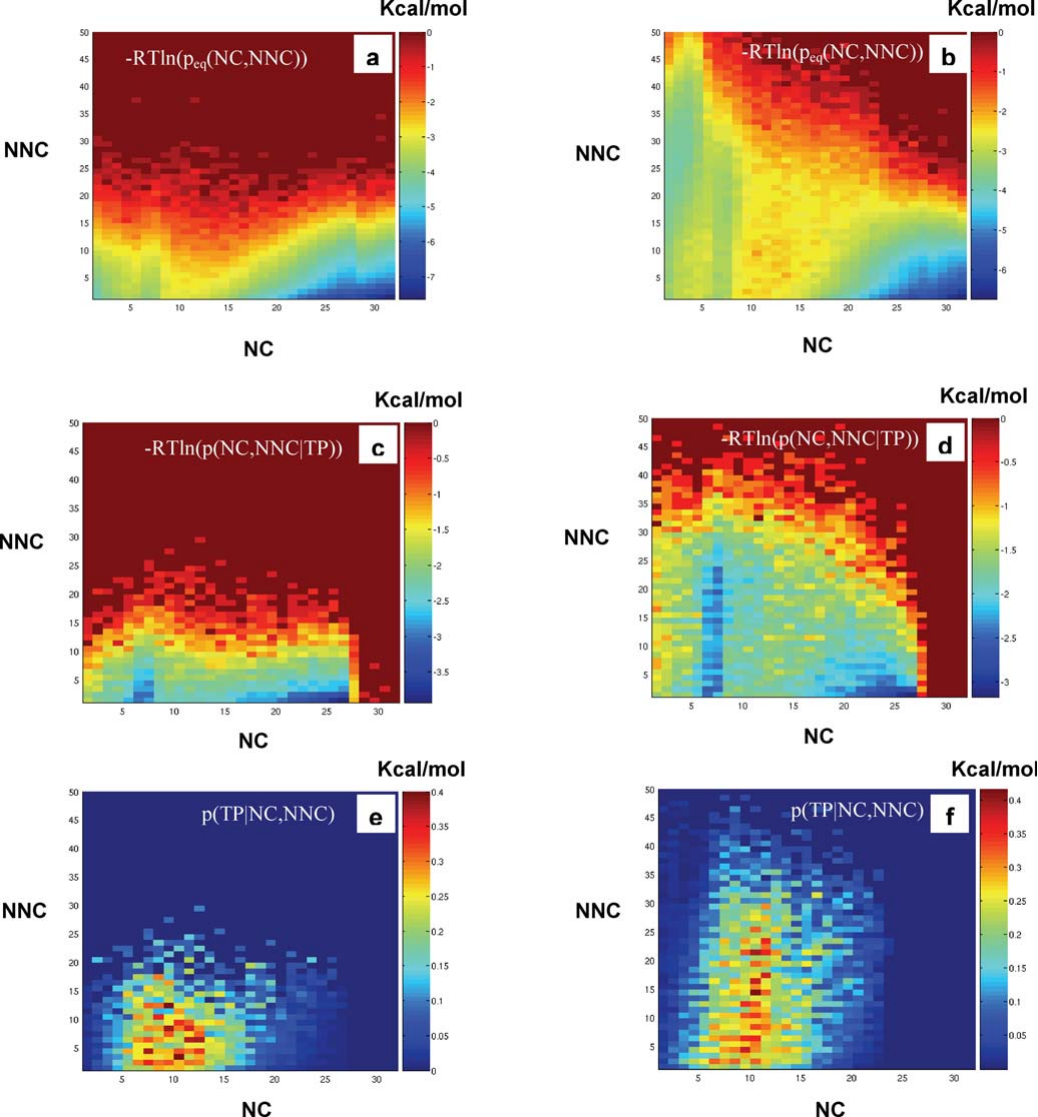
Non-specific encounter complexes. Potential of mean force as function of the number of native and non-native contacts between pKID and KIX. Free energy surfaces are shown as a function of all non-native contacts (NNC) and native contacts (NC) (A–B) at equilibrium and (C–D) in transition paths. (E–F) The probability p(TP|NC,NNC) of being in the transition path as a function of NC and NNC. (A), (C), and (E) correspond to a potential with only native attractive interactions and (B), (D), and (F) correspond to the same potential with added non-native interactions at a strength of 40% of the native contacts.

To clarify the role of non-native binding, as stated above, we performed additional simulations using non-Gō potentials. As discussed, the inclusion of non-native contacts at reasonable strengths does not substantially affect the binding mechanism (Figure S1). However, the additional non-native interactions result in the formation of non-specific encounter complexes. To quantify their population, we determined the binding free energy surface as functions of NNC and NC, the numbers of non-native and native contacts between pKID and KIX, respectively. The results are shown in Figure 7A and 7B for non-native interaction strengths of 0% and 40%, respectively, and in Figure S2 for other interaction strengths.

To explore the role of the non-native interactions in the binding mechanism we calculated the binding free energy surface as a function of NNC and NC selectively for the ensemble of transition paths that connect unbound to bound conformations. Figure 7C and 7D show that substantial non-native interactions are formed during the transition paths with 40% non-native interaction strength, resulting in a free energy minimum for NC∼6 and NNC<25. However, the presence of a minimum does not in itself show whether the non-native interactions are essential elements of the transition state for binding.

We quantify the role of NNC in the binding transition state by calculating the probability p(TP|NC,NNC) of being on a transition path for given values of NC and NNC. Figure 7E and 7F show that the TS for binding, determined by the vertical ridge with p(TP|NC,NNC)∼0.5 (which is the theoretical optimum), is at NC∼10 independent of NNC. Therefore, even though the TS configurations typically have a substantial number of non-native interactions, the binding mechanism is not substantially changed upon the increase of non-specific binding. With the p(TP|NC,NNC)∼0.5 ridge leaning slightly to the right, a larger number of NNC's at the TS appears to require a larger number of NC's as well.

To characterize the nature of the *on-pathway* non-native contacts, reflected in the local minimum in Figure 7D (NC∼6, NNC<25), we calculated the fractions of intermolecular contacts made by each helix (Q_CA_, Q_CB_) and the fraction of intramolecular contacts for each helix (Q_αA_, Q_αB_) in this minimum (Figure S3). The distributions of Q_CA_ and Q_CB_ indicate that in the *on-pathway* complexes α_B_ is partially bound to KIX, with α_A_ mostly dissociated. These conformations have an unstructured α_B_ region that is bound to the hydrophobic groove formed between helices α_1_ and α_3_ of KIX, resembling the binding TS ensemble (Figure 5), but with slightly fewer contacts between α_B_ and KIX (Q_CB_).

We studied also the non-specific binding to the two major solvent exposed surfaces of KIX remote from the native interface (SF1 and SF2 as shown in Figure S4). SF2 includes most of the region previously reported to bind the transactivation domain of the mixed myeloid leukaemia protein (MLL) [40],[41] and recently reported to be involved in transient encounter complexes with pKID [23]. The free energy surfaces as function of the number of non-native contacts between SF1 or SF2 with pKID (NNC_SF1_ and NC_SF2_ respectively) for equilibrium simulations and transition paths are shown in Figure S4. Interestingly, for the MLL binding site SF2 we observe a considerable number of interactions with pKID, in agreement with the NMR results [23]; moreover these transient complexes also have a small number of native interactions, as has been deduced from experiment [23]. While being partially bound to the non-native solvent exposed surfaces of KIX, pKID is thus found to explore other surfaces, including the native interface. This observation is not entirely surprising because pKID is unstructured and KIX is a small protein. However, according to our simulations, SF1 and SF2 do not seem to be involved in the binding pathway towards the native conformation. In the respective free energy surfaces for the transition path ensemble (Figure S4B and S4D), there are no non-native interactions being formed on-pathway with either SF1 and SF2. How can this be reconciled with the NMR results reported by Sugase et al. [23]? The NMR evidence for the location of the encounter-complex binding comes from measurements at 300 µM pKID (MLL titration in Figure 1 of [23]) and ∼1 mM pKID (pKID titration in Figure S2 of [23]). Those concentrations are much higher than the *K_d_* = 1.3 µM [23] of the specific pKID:KIX complex, and it seems likely that under those conditions two pKID molecules could be bound to KIX. The second molecule may form a low affinity complex at the MLL binding site, which has been shown to bind another transcription factor and has high propensity to form non-native contacts with pKID (Figure S4C) [40].

Encounter complexes are thought to be important factors in the folding and binding kinetics [24],[30]. To quantify this effect, we compared the on rate constants (*k_on_*), estimated from mean first passage times, obtained from simulations with non-native interactions strengths of 0% and 40%. The observed ∼4-fold increase in *k_on_* could be explained in terms of an enlargement of the interaction surface, as has been previously shown for globular proteins [27]. Increasing the non-native interaction strength further will eventually produce a slowdown in the rate of specific binding, with non-specific encounter complexes acting as kinetic traps [30].

### Induced-Fit Binding

Molecular complexes in which a “ligand” induces large changes in a “receptor” upon binding have conventionally been described by the “induced-fit” model [42]. Recently, a new view has challenged this simple picture, in which the ligand selectively binds and stabilizes a particular conformation of the receptor that is present at an appreciable population in the equilibrium unbound state [43],[44]. In this “pre-equilibrium” picture, the bound conformation of the “receptor” is formed prior to binding, and “ligand binding” just causes a population shift. Here, it happens to be the “ligand” pKID that undergoes a structural change upon binding, but the above concepts are nevertheless relevant. Our analysis of the transition states shows that structure is formed only after binding, which makes pKID binding to KIX more consistent with an induced-fit interpretation, with the interesting difference that here it is the ligand that changes conformation.

It is easy to see why a population shift mechanism is disfavored in the pKID/KIX transcription factor binding. Stabilization of the folded and bound conformation of pKID comes primarily from the large binding interface with KIX, rather than the relatively weak intramolecular interactions, making the folded conformation, as seen in the bound state, extremely improbable in the unbound state. Although both the induced fit and population shift models originally considered transitions between different conformations of already-folded proteins, the unbound conformations of folded proteins are frequently highly flexible and could be considered analogous to the unfolded peptide here. We stress that both mechanisms are possible in general: the distinction between them in a particular case would depend on the population of the bound conformation in the unbound state.

### Conclusions

The phosphorylated KID domain (pKID) of the transcription factor CREB is known to be unstructured when it is not bound to a target, suggesting that folding and binding are concomitant [11]–[13],[43],[44]. The results of our simulations suggest that binding indeed occurs together with folding. However, we show in addition that folding into a two-helix structure occurs mostly after binding, with the transition state structure of pKID closely resembling the unfolded state.

This mechanism is different from models of protein complex formation in which structure is required for recognition prior to binding. Why would nature adopt such a model for molecular recognition? As mentioned above, there are many advantages to being unstructured, including the capacity to bind multiple targets and rapid termination of the signal *via* degradation of the peptide; since high affinity binding is not required, or even desirable for fast response, pre-existent structure may not be needed. This is also consistent with our finding that the bound complex is relatively loose and includes multiple bound structures.

Nonetheless, despite the acceleration of signal termination, one might imagine that the penalty for being unstructured would be a reduction in the rate of binding, and signal initiation. In fact, we find the opposite to be true: increasing the amount of structure in the unbound pKID slightly reduces the on rate. This slow-down can be understood in the context of the so called “fly-casting” mechanism [32], in which the increased “capture radius” of an unfolded polypeptide enhances the rate of target binding relative to that of compact folded proteins. While the acceleration that we observe, a factor of ∼1.6, would probably not provide a significant advantage biologically, it demonstrates that being unstructured is certainly not a disadvantage from the point of view of binding rate.

A recently published experimental study broadly confirms our simulation results [23]. The main conclusion, of both the experimental study and our simulation study, is that pKID becomes structured only after committing to binding. By performing ^15^N relaxation-dispersion experiments, Sugase et al. [23] inferred that the coupled folding and binding transition involves the formation of an intermediate. Remarkably, this result is also in agreement with our observation of partially bound intermediates states as shown in Figures 3 and 4; we are also able to rationalize the differences in fitted rate coefficients between the two helices based on our simulations.

Non specific transient encounter complexes have been proposed to play an important role in binding of globular proteins [24]–[26],[45]. Kinetic data on a number of protein–protein associations have provided evidence for the initial formation of pre-equilibrium encounter complexes that subsequently relax to the final stereospecific complex [46]. Recently, Sugase et al. [23] found evidence for non-specific transient encounter complexes for the unstructured pKID upon binding to KIX, suggested to be on-pathway based on structural similarities inferred from chemical-shift correlations. Here, we found both on and off-pathway encounter complexes with non-specific interactions. However, we could show that the binding transition state, and thus the binding mechanism, was largely determined by the formation of specific native-like interactions. Nevertheless, non-specific interactions were found to enhance the on-rate for binding, consistent with the results for binding of globular proteins and protein folding [27],[30],[47].

In conclusion, our results support a model for binding of pKID to KIX in which the structure of pKID is induced by binding, conferring several advantages. The importance of unstructured or flexible regions in proteins is increasingly recognized on the basis of both experimental [1], [4], [48]–[50] and theoretical studies [3], [32], [51]–[57]. Since many proteins involved in cell signaling, including transcription factors other than CREB [15], [58]–[63], also have unstructured regions that undergo a transition from unstructured to structured, the mechanism observed here may thus be of more general significance in their target recognition.

## Methods

### Model

The folding and binding of the pKID/KIX complex was studied by using a coarse-grained, native-topology based protein model. The Gō-type model was constructed from the structure of the complex, as determined by NMR (PDB code 1KDX [12]), following a standard procedure [64]. Only residues shown by the NMR data to be structured were used in building the models. The resulting energy surface consists of a transferable dihedral potential, a harmonic potential for angles with a minimum at the native angle, an amino-acid-type dependent (Miyazawa-Jernigan) attractive term [65] for interactions between pairs of residues which interact in the experimental structure (native contacts) and a repulsive potential for all other residue pairs. Each residue is represented by a single bead centered on its alpha-carbon (C_α_) position. To account for the charge of the phosphoserine, amino-acid contact potentials for a glutamic acid were used instead. We scaled the nonbonded interactions in order to have similar populations of bound and unbound conformations. In simulations in the absence of KIX, the α_A_ helix of isolated pKID is only partially formed, and the α_B_ region is mostly unstructured, consistent with previous NMR observations [13].

To sample possible non-native complexes, we generated a model in which attractive potentials were added to all non-native residue pair interactions between pKID and KIX. We used a 12-6 potential with a minimum radius σ = 5.5 Å, and a well depth weighted by the same Miyazawa-Jernigan contact energies as in the Gō-type model, but scaled to 20%, 30%, 40% or 50% of the native value. The interactions of native residue pairs were left unchanged.

### Simulations

A 1:1 complex of pKID/KIX was simulated in a 60 Å cubic box with periodic boundary conditions. Non-bonded interactions were smoothly cut off at 25 Å. The Langevin dynamics simulations were run in CHARMM [66]–[68] with a time step of 15 fs. All bonds were fixed at their native lengths using SHAKE [69]. The actual total simulation time is 195 µs (1.3×10^10^ steps). The chosen friction coefficient of 0.1 ps^−1^ is smaller than that used to mimic water (50–100 ps^−1^) to increase the rate of transitions in the equilibrium simulations [70]. Although these simulations are not in the high friction limit, we can estimate, as a rough approximation, an upper bound for the times in water by scaling simulation times by a factor of 1000 [71], bringing the effective total simulation time to ∼200 ms. We note further that to enhance the sampling of binding events, the binding constant is smaller than the experimental value; however, we find that increasing the affinity by an order of magnitude through enhanced inter-molecular interactions does not affect the results. Simulations with the modified Gō model that includes non-native interactions were run in the same conditions as with the native model. In this case we ran 1.0×10^9^ steps, equivalent to a simulation time of 15 µs, for each of the different potentials.

## Supporting Information

Figure S1 2D free energy surface for different non-native interactions strength. Potential of mean force for binding as a function of the fraction of intermolecular native contacts Q_CA_ and Q_CB_ for potentials with non-native interaction strengths of 20%, 30%, 40%, and 50% of the native ones.
(1.49 MB TIF)

Figure S2 Quantifying the role of non-native interactions. Potential of mean force for binding as a function of the number of intermolecular native contacts (NC) and non-native contacts (NNC) for potentials with non-native interaction strengths of 0%, 20%, 20%, and 50% of the native ones.
(1.15 MB TIF)

Figure S3 On-pathway non-specific binding. (A) The free energy surface as a function of all non-native contacts (NNC) and native contacts (NC) between pKID and KIX in transition path for 40% non-native interaction strength relative to native interactions where the minimum for on-pathway transient complexes is marked. (B) The probability density function of intramolecular native contacts of helix αA (Q_αA_) and helix αB (Q_αB_) of pKID and of native contact fractions Q_CA_ and Q_CB_ between KIX and helices αA and αB of pKID for the selected minimum.
(0.89 MB TIF)

Figure S4 Role of KIX surfaces not involved in the specific pKID/KIX complex. Free energy surfaces are shown as a function of: non-native contacts of pKID with surface 1 of KIX (NNC_SF1_) during (A) equilibrium simulation and (B) transition paths respectively; non-native contacts of pKID with surface 2 of KIX (NNC_SF2_) during (C) equilibrium simulation and (D) transition paths respectively. Regions SF1 and SF2 are illustrated on the structures to the right of (B) and (D), respectively. The potential has 40% non-native interaction strength relative to native interactions.
(1.50 MB TIF)
