# Supplementary figures and images for: Binding-Induced Folding of a Natively Unstructured Transcription Factor
Source: PLoS Comput Biol. 2008 Apr 11;4(4):e1000060. doi: 10.1371/journal.pcbi.1000060 (PMC2289845; doi:10.1371/journal.pcbi.1000060)

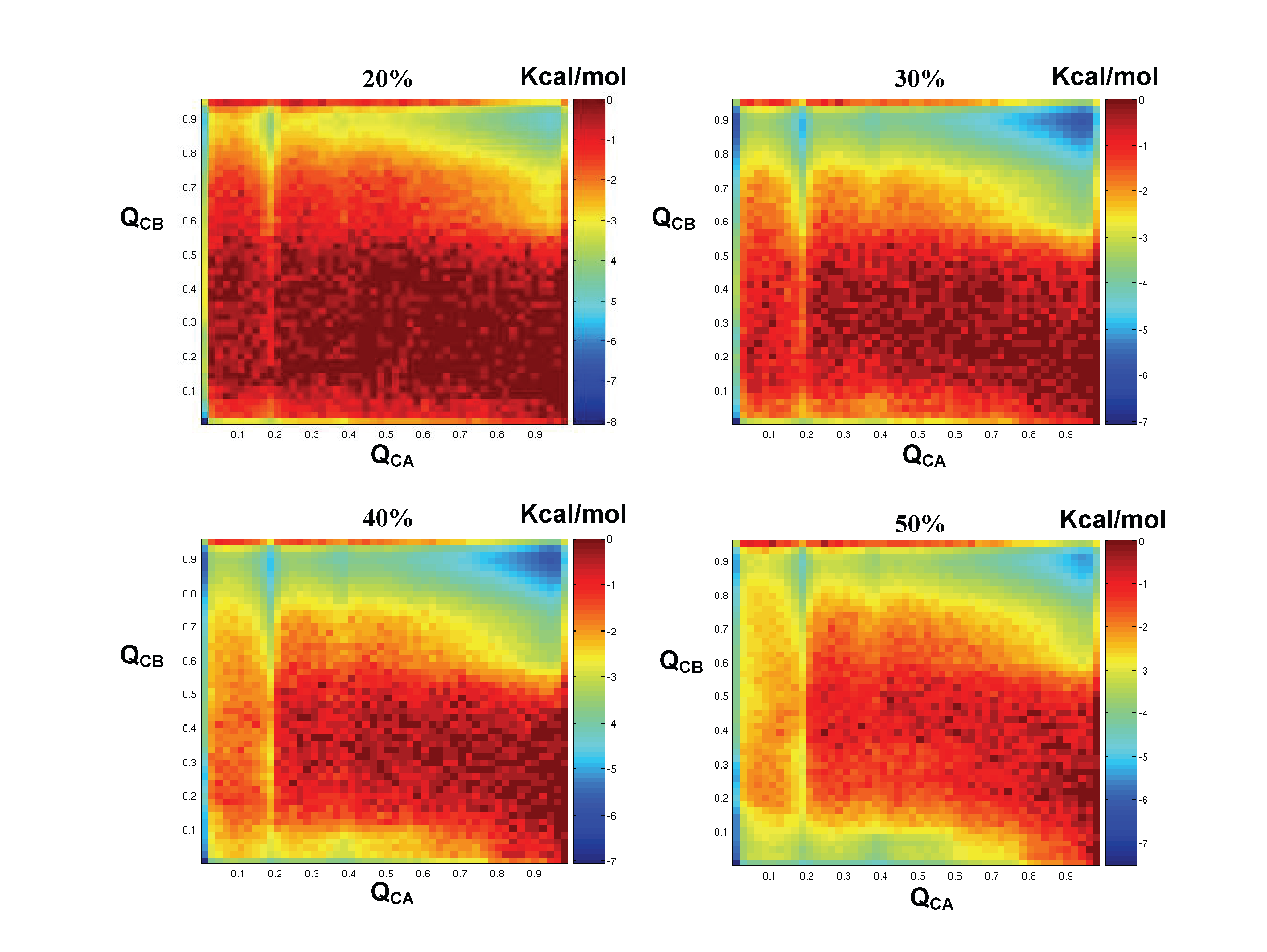

Supplement: Figure S1 — 2D free energy surface for different non-native interactions strength. Potential of mean force for binding as a function of the fraction of intermolecular native contacts QCA and QCB for potentials with non-native interaction strengths of 20%, 30%, 40%, and 50% of the native ones. (1.49 MB TIF) [file pcbi.1000060.s001.tif]

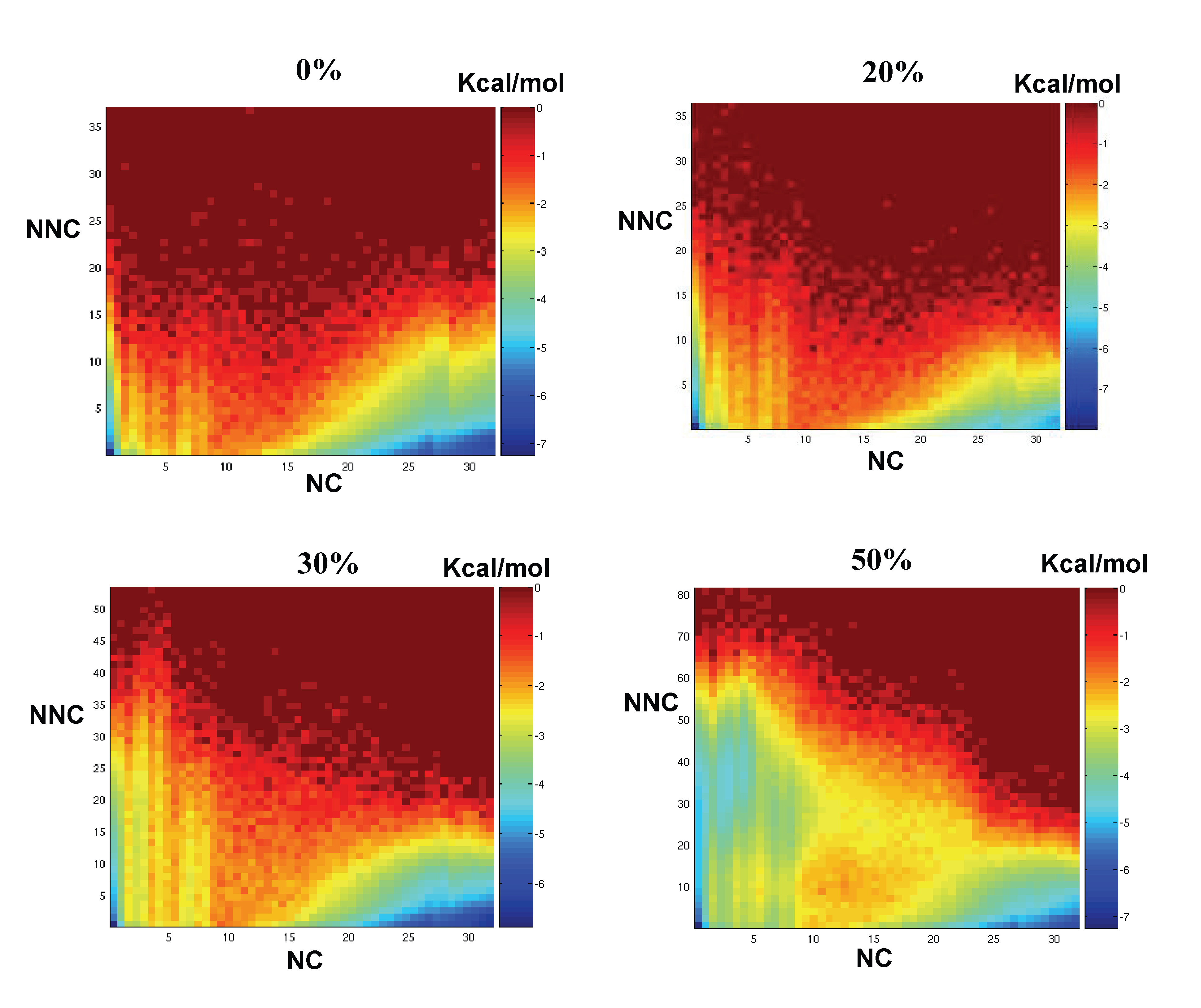

Supplement: Figure S2 — Quantifying the role of non-native interactions. Potential of mean force for binding as a function of the number of intermolecular native contacts (NC) and non-native contacts (NNC) for potentials with non-native interaction strengths of 0%, 20%, 20%, and 50% of the native ones. (1.15 MB TIF) [file pcbi.1000060.s002.tif]

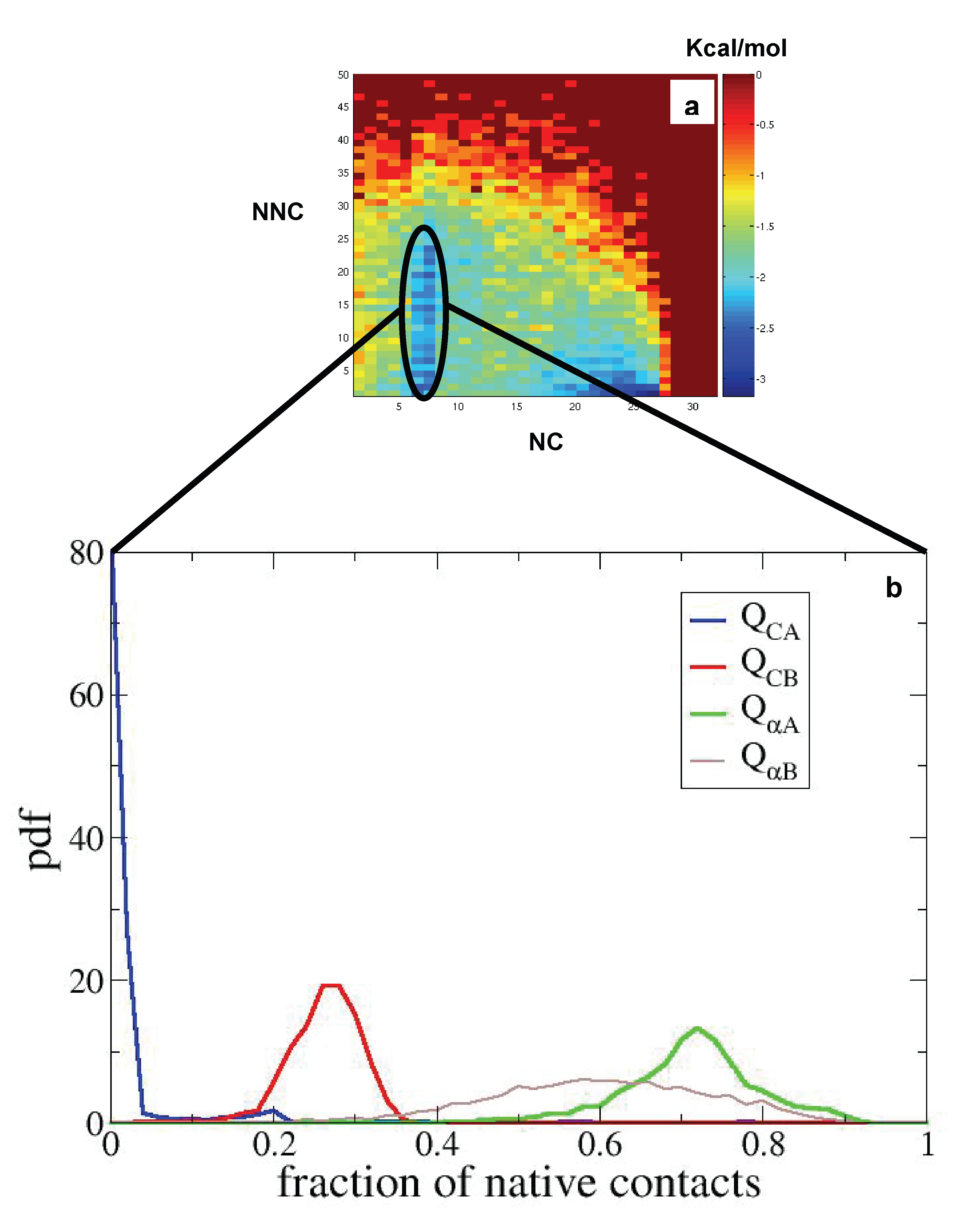

Supplement: Figure S3 — On-pathway non-specific binding. (A) The free energy surface as a function of all non-native contacts (NNC) and native contacts (NC) between pKID and KIX in transition path for 40% non-native interaction strength relative to native interactions where the minimum for on-pathway transient complexes is marked. (B) The probability density function of intramolecular native contacts of helix αA (QαA) and helix αB (QαB) of pKID and of native contact fractions QCA and QCB between KIX and helices αA and αB of pKID for the selected minimum. (0.89 MB TIF) [file pcbi.1000060.s003.tif]

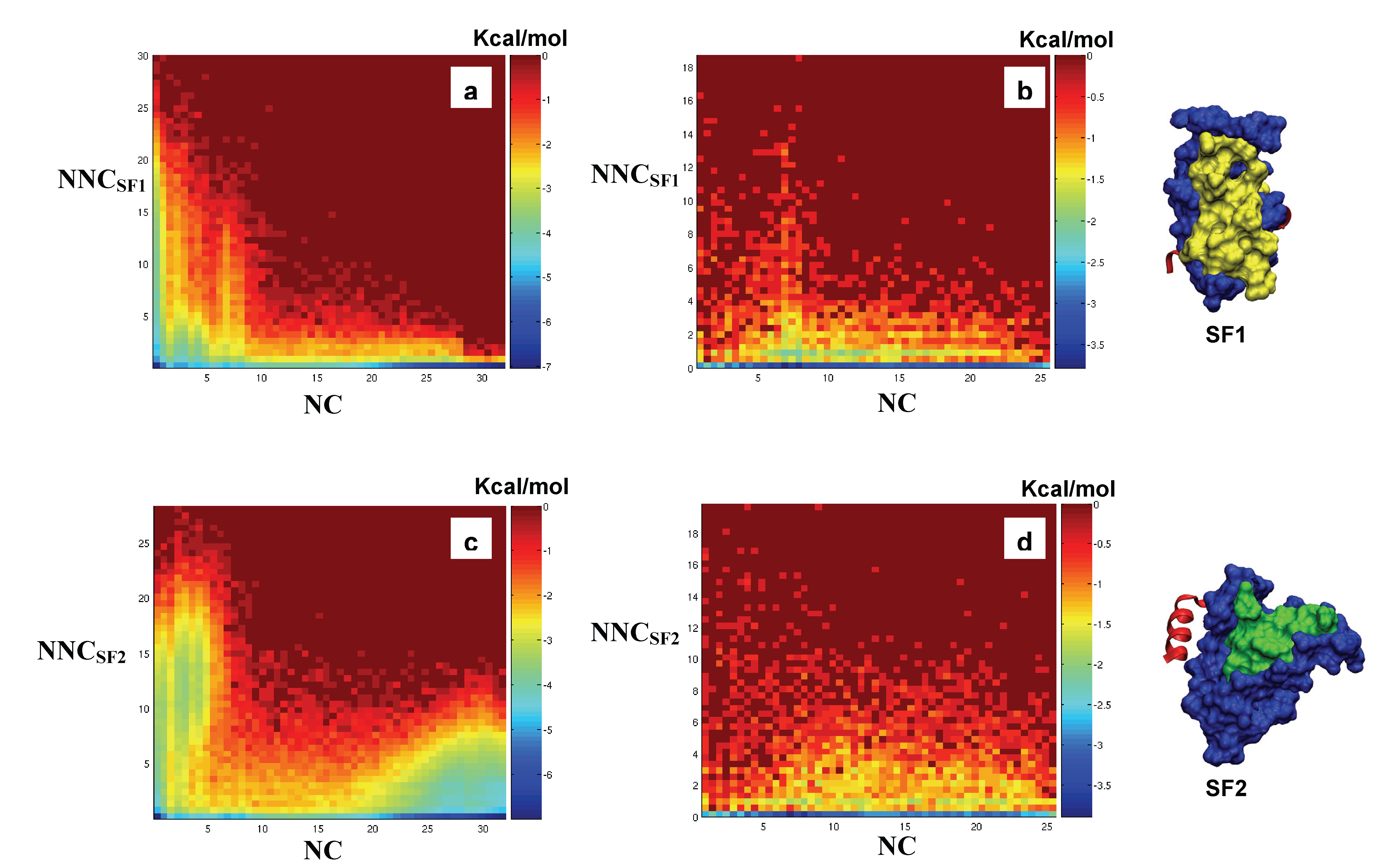

Supplement: Figure S4 — Role of KIX surfaces not involved in the specific pKID/KIX complex. Free energy surfaces are shown as a function of: non-native contacts of pKID with surface 1 of KIX (NNCSF1) during (A) equilibrium simulation and (B) transition paths respectively; non-native contacts of pKID with surface 2 of KIX (NNCSF2) during (C) equilibrium simulation and (D) transition paths respectively. Regions SF1 and SF2 are illustrated on the structures to the right of (B) and (D), respectively. The potential has 40% non-native interaction strength relative to native interactions. (1.50 MB TIF) [file pcbi.1000060.s004.tif]
